# Development of diabetes mellitus associated with quetiapine: A case series

**DOI:** 10.1097/MD.0000000000005900

**Published:** 2017-01-20

**Authors:** Hideki Nanasawa, Akahito Sako, Tomohiko Mitsutsuka, Kaori Nonogaki, Tadayuki Kondo, Shuichi Mishima, Yoriyasu Uju, Toshihiko Ito, Tetsuro Enomoto, Tatsuro Hayakawa, Hidekatsu Yanai

**Affiliations:** a Department of Internal Medicine; b Department of Psychiatry, Kohnodai Hospital, National Center for Global Health and Medicine, Ichikawa, Chiba, Japan.

**Keywords:** antipsychotic drugs, diabetes mellitus, dyslipidemia, quetiapine, schizophrenia

## Abstract

We aimed to describe the characteristics and clinical course of patients who developed diabetes associated with the use of quetiapine.

This study included patients who received quetiapine for over a month between April 2008 and November 2013, and were diagnosed as having new-onset diabetes after initiation of quetiapine. We excluded patients who developed diabetes more than 1 year after discontinuation of quetiapine. We identified new-onset diabetes by hemoglobin A1c or prescriptions of antidiabetic drugs.

Among 1688 patients who received quetiapine, hemoglobin A1c had been measured in 595 (35.2%) patients at least once during the observation period, and 33 (2.0%) patients had received hypoglycemic drugs. Eighteen (1.1%) patients were considered to have developed new-onset diabetes associated with quetiapine after a median of 1.6 years following initiation of quetiapine. Median (interquartile range) age was 54.5 (29.8) years, 8 patients were male, and median (interquartile range) duration of mental illness was 15.3 (13.8) years. Median hemoglobin A1c and body mass index (BMI) were 7.1 (1.4) % and 28.4 (7.0) kg/m^2^, respectively. Seventeen patients had dyslipidemia when diabetes was discovered. All of these discontinued quetiapine within 3 months after the diagnosis of diabetes, and the diabetes in 4 patients had ameliorated without hypoglycemic drugs. Of 13 patients who had received either oral hypoglycemic drugs or insulin, 2 patients achieved well-controlled hemoglobin A1c without hypoglycemic drugs, and 10 patients had hemoglobin A1c 5.0% to 7.7% with the continued use of hypoglycemic drugs.

We demonstrated that almost all patients who developed quetiapine-associated diabetes had dyslipidemia and increased BMI. There was no life-threatening hyperglycemia and diabetes was ameliorated just by discontinuation of quetiapine in several patients. The monitoring of metabolic parameters during antipsychotic treatment is important to diagnose and treat diabetes earlier.

## 1 Introduction

Patients with schizophrenia are at risk of metabolic disorders such as weight gain, hyperglycemia, and diabetes mellitus.^[1]^ Previous studies have shown the relationship between these metabolic disorders and a sedentary lifestyle or unhealthy diet. Genetic factors are also associated with glucose intolerance as shown in a study of drug-naïve patients with schizophrenia and in a study of the genetic relationship between schizophrenia and type 2 diabetes.^[2,3]^

Newer antipsychotic medications have been widely accepted in clinical use because of fewer side effects such as extrapyramidal symptoms.^[4]^ However, it has been observed that these atypical antipsychotics could lead to metabolic dysfunction including diabetes mellitus.^[5,6]^ Previous studies have shown that the rate of diabetes in patients with schizophrenia was 6.2% to 8.7% compared with 1.1% in males without schizophrenia.^[7]^ A large-scale observational study using an administrative database showed that 18% of patients with schizophrenia who received antipsychotics also had a diagnosis of diabetes. Moreover, patients who received atypical antipsychotics had significantly higher likelihood of developing diabetes than those who received typical antipsychotics (odds ratio: 1.09; 95% confidence interval: 1.03–1.15).^[8]^

Quetiapine, a second-generation antipsychotic drug, is commonly used for the treatment of schizophrenia. Previous studies have shown that quetiapine also induced weight gain, hyperlipidemia, and diabetes, similarly to other atypical antipsychotics such as olanzapine and clozapine which exert more influence on glucose tolerance.^[9,10]^ In contrast, it has been reported that the risk for diabetes caused by quetiapine was not significantly different from that caused by conventional antipsychotics.^[11,12]^ Patients with diabetes in Asia are characterized by onset at a relatively younger age and lower BMI compared with those in Western countries.^[13,14]^ The mechanisms are explained by unstable life-energy balance or increased insulin resistance. Persons of Asian descent have a greater amount of visceral adipose tissue than Europeans with the same BMI or waist circumference.^[15]^ Asian Indians living in the United States were found to have both greater insulin resistance and higher plasma high-sensitivity C-reactive protein than Caucasians.^[16]^ Although race and ethnicity are associated with characteristics of diabetes, little is known about quetiapine-induced diabetes in the Asian population.^[17,18]^

We sought to describe the characteristics, metabolic complications, and consequences of diabetes in Japanese patients treated with quetiapine primarily for schizophrenia and mood disorders.

## 2 Methods

### 2.1 Hospital setting

This retrospective descriptive case series study was conducted at Kohnodai hospital, National Center for Global Health and Medicine, an acute care general hospital located close to Tokyo. In 2014, the hospital had an average of 300 inpatients and 810 outpatients daily. Because this hospital was formerly the National Center for Psychiatry and Neurology, approximately one-third to half of inpatients and outpatients were seen in the department of psychiatry. In accordance with the ethical guidelines for medical and health research involving human subjects in Japan, informed consent from each patient was not required for this retrospective, observational, noninterventional study, and we placed posters around the hospital describing the study and the ability to opt out if they so desired. The study protocol was approved by the Ethics Committee of the National Center for Global Health and Medicine (NCGM-G-001518).

### 2.2 Patients

Patients were screened in 2 steps. First, using the clinical research database in the hospital, we retrospectively extracted patients who had received quetiapine at least once between April 2008 and November 2013 and had been diagnosed as having diabetes. We extracted patients irrespective of whether they were inpatients or outpatients. We identified patients with possible diabetes by a hemoglobin A1c (HbA1c) level of 6.5% or greater, or prescriptions for antidiabetic drugs. Because we could not tell whether plasma glucose was fasting or postprandial, screening of diabetes was based on HbA1c or prescription for diabetes. In Japan, antidiabetic drugs are approved only for the treatment of diabetes and hyperglycemia and not for other metabolic diseases. Second, we reviewed each patient's clinical records and considered the patient as having quetiapine-induced diabetes based on following criteria: having received quetiapine for over a month, having developed new-onset diabetes after initiation of quetiapine, having developed new-onset diabetes during quetiapine treatment or within 1 year after discontinuation of quetiapine. We confirmed the diagnosis of diabetes based on the diagnostic criteria as recommended by the Japanese Diabetes Society, in which the cut-off values for fasting plasma glucose, casual plasma glucose, and HbA1c were ≥126 mg/dL (≥7.0 mmol/L), ≥200 mg/dL (≥11.1 mmol/L), and ≥6.5%, respectively.^[19]^

### 2.3 Variables of interest and study outcomes

We retrospectively collected data before, during, and after quetiapine treatment, from a clinical database and medical records. After the screening, we investigated age, gender, body weight, body height, duration of psychiatric illness, medication including olanzapine, clozapine, corticosteroids and β-stimulants, medical history and familial history of metabolic disorders, laboratory tests including plasma glucose, HbA1c, total cholesterol, high-density lipoprotein-cholesterol, low-density lipoprotein-cholesterol (calculated by Friedewald formula or measured directly), triglycerides, and urine acid, as well as the clinical course of diabetes. BMI was calculated using the following standard equation: BMI = weight in kilograms/height squared in meters. We did not investigate symptoms and dysfunction associated with mental disorders because obtaining such information in a retrospective study was difficult. Data were expressed as median (interquartile range).

## 3 Results

**Figure 1:**
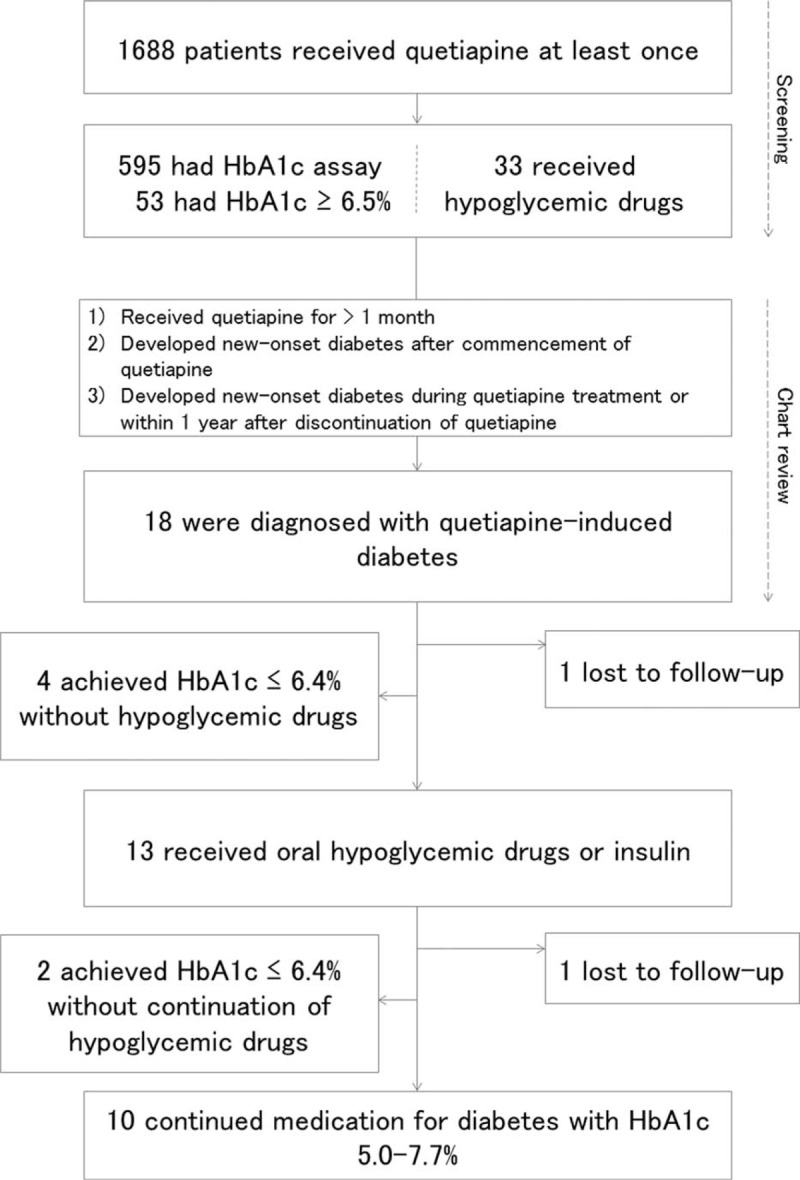
Flow diagram showing eligible patients and the clinical course of diabetes.

A total of 1688 patients in the database were identified as having received quetiapine (Fig. 1). Of these, HbA1c had been measured in 595 (35.2%) patients at least once during the observation period. As a result of screening, 53 (3.1%) patients had HbA1c ≥6.5 and 33 (2.0%) patients had received hypoglycemic drugs. After reviewing the clinical records, we excluded patients who had been diagnosed as having diabetes before initiation of quetiapine, or who did not meet the diagnostic criteria for diabetes, or those in whom quetiapine did not induce diabetes. Eighteen (1.1%) patients were eligible based on the criteria and were considered to have new-onset diabetes associated with quetiapine (Tables 1 and 2). They developed diabetes a median of 1.6 (2.2) years after starting quetiapine. The median age at the time of diagnosis of diabetes was 54.5 (29.8) years, 8 cases were male, and 10 cases were female. Eleven patients had a diagnosis of schizophrenia, 3 had mood disorders such as major depression or bipolar disorder, and 4 had other mental disorders. Median duration of mental illness was 15.3 (13.8) years and median dose of quetiapine was 100 (150) mg just before discontinuation. Median HbA1c was 7.1 (1.4) %. Median body weight and BMI were 68.9 (23.8) kg and 28.4 (7.0) kg/m^2^, respectively. Five patients had used other atypical antipsychotic drugs with quetiapine and 3 had used olanzapine. No patients had been prescribed systemic corticosteroids. At the time of diagnosis of diabetes, 17 patients had dyslipidemia and 10 had been treated with lipid-lowering agents. Eight patients had hypertension and 7 had been treated with anti-hypertensive drugs. Four patients had hyperuricemia but had received no medication. These metabolic parameters deteriorated after initiation of quetiapine and were ameliorated after quetiapine discontinuation.

**Table 1:**
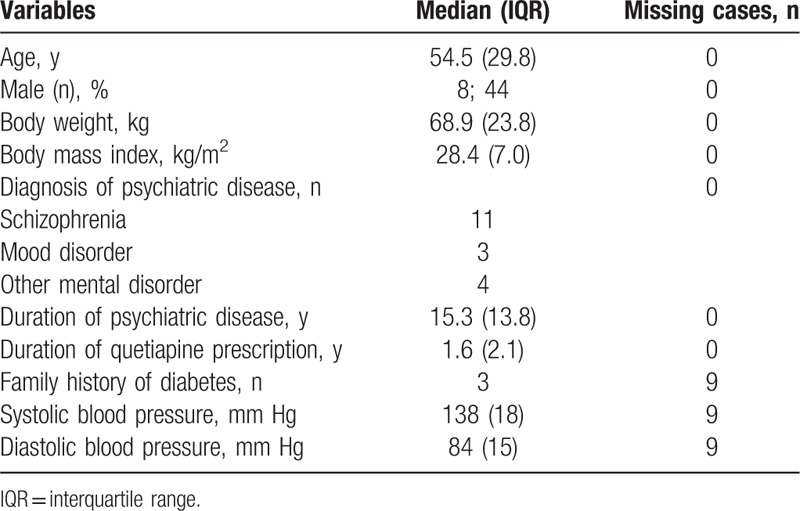
Background characteristics of patients at diagnosis of diabetes.

**Table 2:**
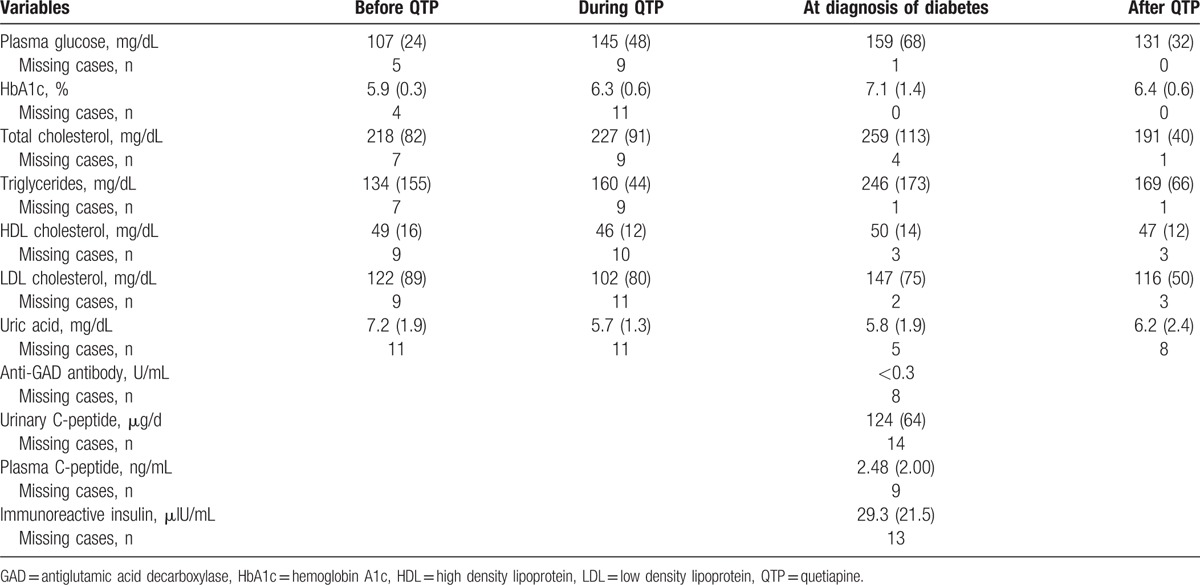
Patient laboratory data.

All of the patients discontinued quetiapine within 3 months after the diagnosis of diabetes, and in 4 patients HbA1c decreased to 5.9% to 6.6% without hypoglycemic drugs. One patient had been diagnosed as having diabetes just before the end of the observation period, thus, we could not follow the clinical course of the disease.

Of the 13 patients who had received either oral hypoglycemic drugs or insulin, 2 patients achieved HbA1c 6.4% or less without hypoglycemic drugs, and 10 patients had HbA1c 5.0% to 7.7% with the continued use of hypoglycemic drugs. One patient did not visit our hospital after the diagnosis of diabetes, and data about clinical course were not available. Five patients were admitted to the department of internal medicine for management of diabetes, however, no patients had life-threatening complications such as diabetic ketoacidosis or diabetic hyperglycemic hyperosmolar syndrome. In addition, antiglutamic acid decarboxylase (GAD) antibody was measured in half of the patients and was not detected in any patient. Defective insulin secretion such as significant reduction of urinary and plasma C-peptide and immunoreactive insulin were not observed. None of the patients required continuous insulin treatment.

## 4 Discussion

We found that 18 patients developed diabetes among 1688 patients who received quetiapine at least once between 2008 and 2013. Most of them had obesity and hyperlipidemia. Of the 13 patients who received either oral hypoglycemic drugs or insulin, 2 patients achieved well-controlled HbA1c and could stop the medication, and 10 achieved HbA1c levels of 5.0% to 7.7% with continued use of hypoglycemic drugs. In 4 patients, diabetes was ameliorated without hypoglycemic drugs.

Existing data show an increased risk for diabetes in patients receiving second-generation antipsychotics.^[4]^ The risk in patients on quetiapine is less clear than clozapine or olanzapine, because some studies showed an increased risk for diabetes, while others did not. Previous studies suggested that mechanisms of weight gain, overeating, and metabolic disorders are mediated by antagonists of multiple receptors especially H_1_ and serotonin 5-HT_2C_ receptors.^[20,21]^ Serotonin is known to play a role in glucose homeostasis, and 5-HT_1A_ antagonism leads to a decrease in insulin secretion secondary to decreased pancreatic β-cell responsiveness to plasma glucose levels.^[5]^ In an experiment with hamsters, insulin-secreting pancreatic β-cell apoptosis induced by olanzapine was observed.^[22]^ However, in our study, no patient needed insulin treatment permanently. Moreover, no patient showed defective insulin secretion or autoimmune diabetes among the patients for whom urinary or plasma C-peptide or immune-reactive insulin, or anti-GAD antibody data were available.

A postmarketing survey of 1158 patients who received quetiapine was conducted in Japan between 2001 and 2003^[23]^ (Table 3). Among 267 nondiabetic patients who underwent HbA1c monitoring before and during quetiapine treatment, 6 patients (2.2%) developed new-onset diabetes. Although overweight, overeating, and hypoactivity were associated with hyperglycemia, other risk factors such as hyperlipidemia and hyperuricemia were not studied. Another prospective cohort study in Japan showed that 5.2% of patients who used second-generation antipsychotics developed diabetes, and hyperlipidemia and a family history of diabetes were risk factors.^[24]^ The results of our study are comparable with these large-scale studies, but the study design was different and we used HbA1c as an index for screening because it was not influenced by the timing of blood tests and meals. Moreover, we included other risk factors such as hyperlipidemia and hyperuricemia.

**Table 3:**
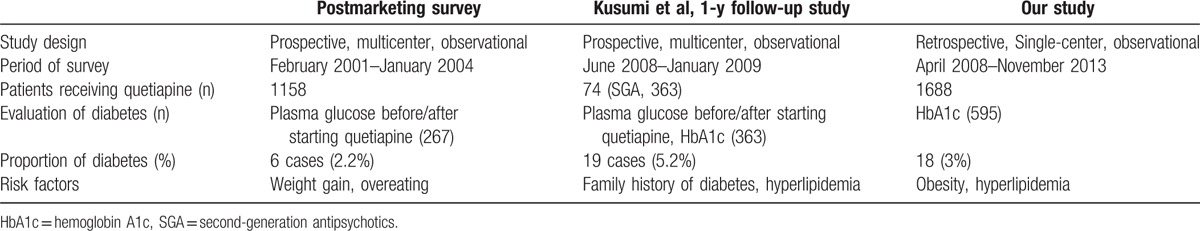
Comparison with previous studies in Japan.

Most large-scale studies focused on the relationship between atypical antipsychotic drugs and onset of diabetes.^[8,25,26]^ In this regard, the postmarketing survey in Japan was conducted similarly, but the clinical course after onset of diabetes remains unclear. We followed the clinical course of diabetes in these patients for approximately 1 year on average and found that there were certain patients in whom diabetes had ameliorated with or without treatment. Recent case reports show that quetiapine-induced diabetes could improve without hypoglycemic drugs after discontinuation of the antipsychotic drugs.^[17,27–29]^

A consensus statement by the American Diabetes Association, American Psychiatry Association, American Association of Clinical Endocrinologists, and North American Association for the Study of Obesity recommends that all patients receiving antipsychotics should receive appropriate baseline screening and ongoing monitoring of whether they have diabetes or not.^[4]^ Although clinicians have tended to monitor plasma lipids and glucose after this report was issued, it is not still enough.^[30]^ We surmise that this is because psychiatrists could not pay attention to metabolic side effects or had difficulty in obtaining patient consent for blood tests. Olanzapine and quetiapine are contraindicated in patients with a current or history of diabetes because of reports of fatal diabetic hyperglycemia associated with these drugs in Japan.^[31,32]^ After these serious observations, Tsubouchi et al^[33]^ reported that approximately 60% patients receiving atypical antipsychotics had not had plasma glucose or HbA1c analyzed within a year, even in a university hospital. In Japan, there is a monitoring guidance for blood glucose in patients being treated with atypical antipsychotic drugs based on a review of consensus guidelines and articles, the characteristics of Japanese patients, and the healthcare environment.^[34]^ To prepare for patients from various backgrounds, we should adhere to such monitoring guidance to enable clinicians to quickly identify patients at risk of metabolic disorders or abnormalities.

There are several limitations of this study. First, some data were missing such as weight gain, smoking status, dietary intake, plasma glucose, or other metabolic markers for some patients because this was a retrospective study. However, available data showed that patients with quetiapine-associated diabetes often had obesity and dyslipidemia. Second, we did not investigate other antipsychotic medications that were used at the same time except for olanzapine and clozapine because other atypical and typical antipsychotics are less likely to cause diabetes.^[4]^ We could not exclude the influence of these concomitant drugs. However, we confirmed that none of the patients had used systemic corticosteroids which were the most common and most significant cause of drug-induced diabetes. Third, we did not investigate patients who did not develop diabetes. Consequently, we could not elucidate the incidence of, and risk factors for quetiapine-associated diabetes. We could not analyze whether the development of diabetes was dose-dependent.

In conclusion, this study provides valuable information on the characteristics and consequence of diabetes in patients who developed diabetes associated with quetiapine. Most patients who developed diabetes were treated with medication and recovered to some extent. Interestingly, in 4 patients, diabetes was ameliorated without hypoglycemic drugs. Further studies are needed to clarify the characteristics of quetiapine-induced diabetes in order to use quetiapine properly and safely.
